# Lewy Body Dementia Association’s Industry Advisory Council: proceedings of the second annual meeting

**DOI:** 10.1186/s13195-021-00868-7

**Published:** 2021-07-08

**Authors:** Jennifer G. Goldman, Bradley F. Boeve, Melissa J. Armstrong, Doug R. Galasko, James E. Galvin, David J. Irwin, James B. Leverenz, Karen Marder, Victor Abler, Kevin Biglan, Michael C. Irizarry, Bill Keller, Robert Lai, Leanne Munsie, Michael Belleville, Ondrea Chaney, Ian Richard, Angela Taylor, Todd Graham

**Affiliations:** 1 Parkinson’s Disease and Movement Disorders, Shirley Ryan AbilityLab and Departments of Physical Medicine and Rehabilitation and Neurology, Northwestern University Feinberg School of Medicine, 355 E. Erie Street, Chicago, IL USA; 2 Department of Neurology, Mayo Clinic, Rochester, MN USA; 3 Department of Neurology, University of Florida College of Medicine, Gainesville, FL USA; 4 Department of Neurosciences, UC San Diego, San Diego, CA USA; 5 Comprehensive Center for Brain Health, Department of Neurology, University of Miami Miller School of Medicine, Miami, FL USA; 6 Department of Neurology, Hospital of the University of Pennsylvania, Philadelphia, PA USA; 7 Lou Ruvo Center for Brain Health, Neurological Institute, Cleveland Clinic, Cleveland, OH USA; 8 Department of Neurology, Taub Institute, Sergievsky Center, Columbia University Irving Medical Center, New York, NY USA; 9 Acadia Pharmaceuticals, San Diego, CA USA; 10 Neuroscience Research, Eli Lilly and Company, Indianapolis, IN USA; 11 Neurology Business Group, Eisai, Inc., Woodcliff Lake, NJ USA; 12 Lewy Body Dementia Association, S.W, Lilburn, GA USA

**Keywords:** Clinical trial, COVID-19, Lewy body dementia, Research, Telemedicine

## Abstract

In 2019, the Lewy Body Dementia Association formed an Industry Advisory Council to bring together a collaborative group of stakeholders with the goal of accelerating clinical research into Lewy body dementia treatments. At the second annual meeting of the Industry Advisory Council, held virtually on June 18, 2020, the key members presented ongoing and planned efforts toward the council’s goals. The meeting also featured a discussion about the effects of the COVID-19 pandemic on Lewy body dementia clinical research, lessons learned from that experience, and how those lessons can be applied to the design and conduct of future clinical trials. This report provides a brief summary of the meeting proceedings with a focus on efforts to improve and adapt future Lewy body dementia clinical research.

## Introduction

Currently available medical strategies for managing Lewy body dementia (LBD) are limited and lack the ability to modify the course of the disease. Major obstacles to the development of better therapies for LBD are the many unanswered questions about clinical trial design in this setting. Such questions include how to define disease subtypes, select enrollment criteria, monitor disease progression, and measure primary, secondary, and exploratory outcomes. With the goal of stimulating clinical research in LBD treatments, in 2019, the Lewy Body Dementia Association (LBDA) formed an Industry Advisory Council (LBDA IAC) comprising a diverse group of academic and industry researchers as well as patient advocacy representatives. Proceedings of the inaugural meeting of the LBDA IAC were published along with detailed descriptions of the challenges being addressed (Goldman et al. 2020). We now report the progress and current activities of the IAC as described at the second annual meeting held virtually on June 18, 2020.

## COVID-19 and clinical trials: lessons for the future

At the time of the IAC meeting, the COVID-19 pandemic had been ongoing in the USA for approximately 4 months with a national emergency declared in March 2020. The IAC members from different pharmaceutical companies described how their respective organizations adapted procedures for ongoing clinical trials in response to the pandemic. This report focuses on the lessons learned that are potentially applicable to the design and execution of future clinical trials in LBD. Major goals were to ensure the safety of patients and investigators, to minimize the risk to trial integrity, and to maintain trial quality. Prompt and regularly updated guidance from the Food and Drug Administration was a key factor in achieving these goals. Patients were able to continue in studies as long as safety and efficacy monitoring could be maintained, though most studies paused screening and enrollment of new participants. In some trial settings, digital and telemedicine tools were already supplanting some in-person tasks, and those pre-existing capabilities helped investigators maintain trial procedures and integrity. Some specific examples included the use of tablets for recording data, cloud-based data storage, centralized and continual data and safety monitoring and source data verification, and training of trial staff using a centralized portal. The COVID-19 pandemic required and accelerated the use of other tools including electronic consent forms, web-based investigator meetings, home visits including home-based infusions, use of alternative laboratories, online community outreach, COVID-19 testing, and especially greater use of telemedicine. Many of these adaptations required protocol changes and approvals by central or local Institutional Regulatory Bodies (IRBs), which was challenging because many IRBs had been instructed to prioritize COVID-19 protocols. The industry members noted that telemedicine visits were feasible and should be considered for routine use in future trials when possible; however, assessment of some LBD-related endpoints is difficult or impossible via telemedicine, and many of the commonly used assessment tools need to be validated for use in the telemedicine setting.

Several notable lessons were learned from how the COVID-19 pandemic affected observational and basic science studies at academic medical centers. Observational studies will need to determine how to handle missing data, how to safely and efficiently re-start studies, and how to proceed when patients or families are reluctant to come for in-person research visits. Basic scientists were challenged with how to maintain critical assets such as cell cultures and animal colonies. In addition, researchers were disrupted by hiring freezes, furloughs of support staff such as grant administrators, requirements to work from home, and the need to care for children or family members. These disruptions have had substantial effects on the collection of preliminary data for grant applications, promotion timelines, and fundraising efforts. IAC members noted that many academic sites were substantially affected due to institution-wide shutdowns, and some experienced over-capacity in clinics; in contrast, many commercially operated trial sites were under-utilized.

At this meeting, a person living with LBD and participating in a clinical trial at the time the pandemic arrived shared his perspective. In this person’s view, many of the changes brought about by the pandemic lockdowns were beneficial for patients and should be continued when possible. Such changes included the ability to go to a local lab to give blood samples, and the ability to have clinic visits done remotely. Traveling to clinics for research visits may be stressful for patients, especially those with dementia, so there was a discussion about how that stress may affect cognitive testing. Further exploration is warranted to improve our understanding of how people in the LBD community use technology. With that understanding, clinical trials can be designed to use remote assessments, which may allow the trials to draw from a much larger population of patients who are reluctant to regularly travel to clinics.

## Clinical trial planning in LBD

The group discussed various efforts of the LBDA Research Centers of Excellence (RCOE) program to address unresolved issues regarding the design of interventional clinical trials in LBD. One major topic was the lack of cognitive assessment tools that are specific for LBD or that address the differences in cognitive decline between Parkinson’s disease dementia and dementia with Lewy bodies. Recent studies on this issue indicate that more longitudinal data are needed to guide clinical trial design. Another major challenge for clinical trials is the longitudinal measurement of cognition and LBD symptoms when those parameters exhibit large fluctuations. Other unresolved issues include how to define optimal disease management (“standard-of-care”) at baseline, which is expected to be an essential component of trials comparing an intervention against a standard-of-care. Industry members also noted trends toward studying narrow, “precision” indications; this trend is expected to rely on biomarkers that indicate specific diagnoses and that can be used to track disease progression. Related discussions focused on the need to identify reliable and valid biomarkers of Alzheimer’s co-pathology in LBD trial participants. Identifying relevant therapeutic targets and demonstrating target engagement is an important consideration for LBD trials. Many efforts such as those focused on alpha-synuclein immunotherapy, LRRK2 inhibitors, glucocerebrosidase mechanisms, and others are starting in Parkinson’s disease rather than DLB, and we await outcomes from these studies to discern how they inform LBD clinical trials.

## Patient needs and patient-reported outcomes

Studies are needed to identify the unmet needs of people living with LBD and their caregivers, and this need is an opportunity for partnerships between academic medical centers and patient advocacy organizations such as the LBDA. As a first step toward addressing that need, the members of our group discussed how structured interviews with people who have LBD and caregivers could help study LBD symptoms and natural history, how LBD affects function and quality of life, experiences with treatment, which outcomes are important, and other issues important to patients and caregivers. These efforts will help identify research gaps, influence clinical trial design and selection of endpoints, and guide regulatory review of new symptomatic or disease-modifying treatments. It is anticipated that this effort will serve as a starting point for more detailed studies leading to the development of robust patient-reported outcome measures for both cognitive and non-cognitive symptoms of LBD. To achieve these goals, collaboration is essential and should include academic, government, and industry researchers; specialists with expertise in treating various LBD symptom; regulatory and funding agencies; individuals with LBD; caregivers; and the LBDA.

In summary, LBD research has adapted to changes during the COVID-19. Clinical trials remain a priority in the field, and identifying key measures, biomarkers, and optimal study designs and establishing partnerships among researchers, regulatory and funding agencies, and the patient community remain essential components.

## Data Availability

NA.

## Abbreviations

IAC: Industry Advisory Council
IRB: Institutional Regulatory Body
LBD: Lewy body dementia
LBDA: Lewy Body Dementia Association
RCOE: Research Center of Excellence
